# Sphincter-saving resection by cluneal arched skin incision for a gastrointestinal stromal tumor (GIST) of the lower rectum: a case report

**DOI:** 10.1186/s40792-016-0285-8

**Published:** 2017-01-05

**Authors:** Hirofumi Tazawa, Yuzo Hirata, Yoshio Kuga, Toshihiro Nishida, Hideto Sakimoto

**Affiliations:** 1 Department of Surgery, Chugoku Rosai Hospital, 1-5-1, Tagaya, Hiro, Kure City, Hiroshima 737-0193 Japan; 2 Department of Internal Medicine, Chugoku Rosai Hospital, 1-5-1, Tagaya, Hiro, Kure City, Hiroshima 737-0193 Japan; 3 Department of Diagnostic Pathology, Chugoku Rosai Hospital, 1-5-1, Tagaya, Hiro, Kure City, Hiroshima 737-0193 Japan; 4 Department of Gastroenterological and Transplant Surgery, Applied Life Sciences, Institute of Biomedical and Health Sciences, Hiroshima University, 1-2-3, Kasumi, Minami-ku, Hiroshima 734-8551 Japan

**Keywords:** Rectal GIST, Cluneal arched skin incision, Sphincter-saving operation

## Abstract

**Background:**

Planning the surgical strategy for a gastrointestinal stromal tumor (GIST) at the posterior wall of the lower rectum is difficult, as the procedures for the lower rectum are hampered by poor visualization and may cause anal dysfunction or discomfort. We report a novel procedure to resect a submucosal tumor of the rectum.

**Case presentation:**

A 75-year-old woman presented with metrorrhagia. Endovaginal ultrasonography showed a low echoic tumor. Computed tomography showed an enhanced tumor, measuring 5.3 × 4.2 cm, behind the rectum. Magnetic resonance imaging revealed a submucosal tumor of the rectum, measuring 5.3 cm at its greatest dimension. Colonoscopy showed that the distal tumor margin was 1 cm above the dentate line. Core needle biopsy of the tumor revealed the rectal GIST. After receiving neoadjuvant imatinib treatment, the tumor size decreased to 3.5 cm. During the operation, we approached the rectum and resected the posterior rectal wall, including the 3.5 × 3.5 cm tumor with a safety margin, making an arched incision at the buttocks to form a skin flap with the patient in a jackknife position. The histopathological diagnosis was GIST of the rectum. Her anorectal sphincter function was well preserved. No recurrence was seen during the 2-year follow-up.

**Conclusions:**

This novel approach improves the operative field visibility in resecting a tumor with a safety margin and preserves a patient’s anorectal sphincter function.

## Background

Gastrointestinal stromal tumors (GISTs) occur most often in the stomach (60%) and the small intestine (35%) [1]. GISTs of the colon and rectum constitute <5% of all cases and occur more often in the rectum [2]. Surgery with histologically negative margins is the recommended primary treatment for nonmetastatic GISTs [3]. Neoadjuvant therapy of imatinib, a selective tyrosine kinase inhibitor, for rectal GISTs, with the aim of preserving the anus, is still a challenging therapy that typically takes several months [4]. Planning the surgical strategy for a GIST at the posterior wall of the lower rectum is difficult, as the procedures for the lower rectum are hampered by poor visualization and may cause anal dysfunction or discomfort. We describe our experience with a patient who underwent a novel approach, which improves the operative field visibility in resecting a tumor with a safety margin and preserves a patient’s anorectal sphincter function.

## Case presentation

**Fig. 1:**
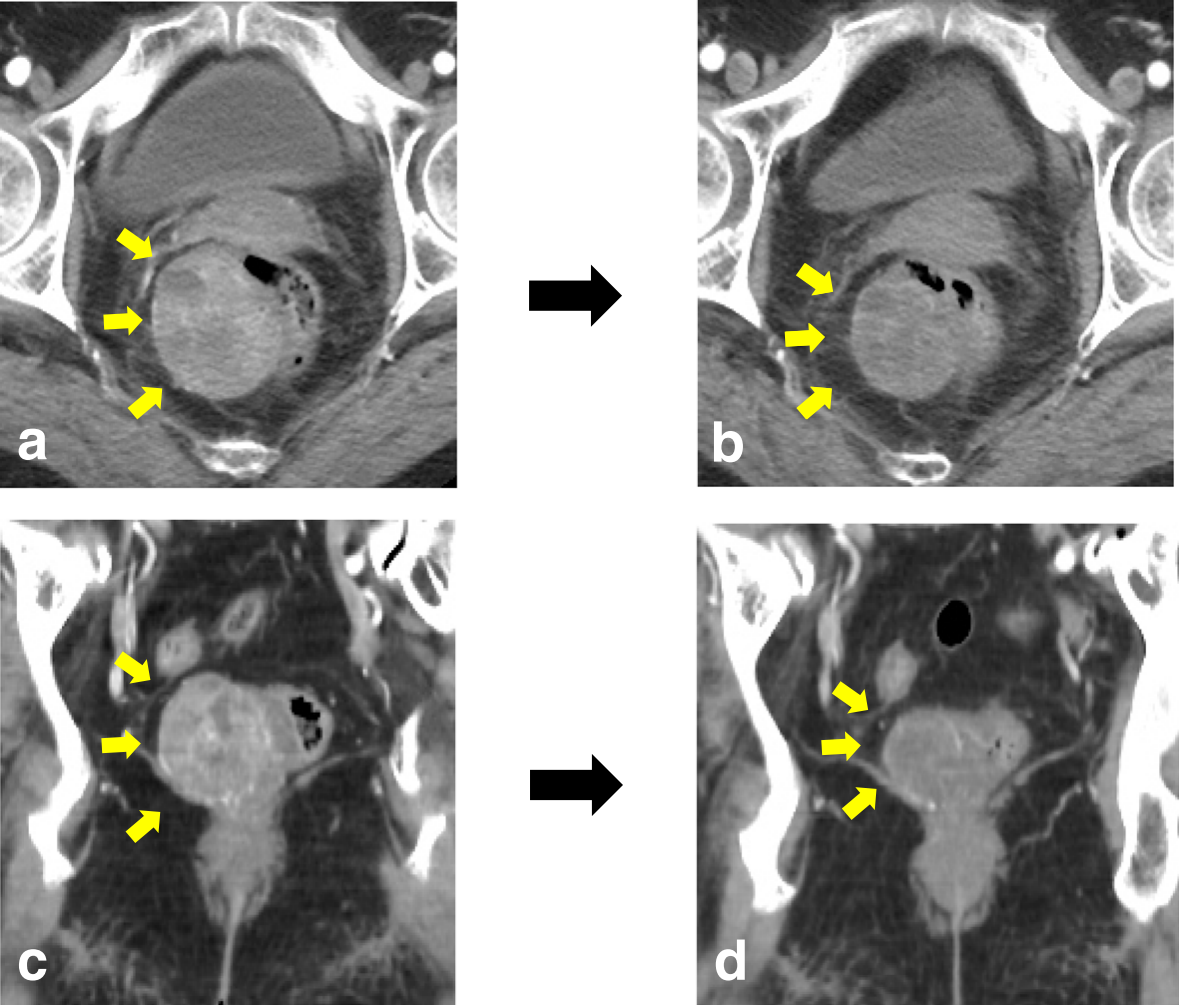
**a** Coronal CT images showed an enhanced tumor measuring 5.3 × 4.2 cm behind the rectum. **b** After the treatment of imatinib, coronal CT images revealed that the tumor size decreased to 3.5 cm. **c** Axial CT images showed an enhanced tumor at the right posterior side of the rectum. **d** After treatment of imatinib, axial CT images revealed that the tumor size decreased to 3.5 cm

**Fig. 2:**
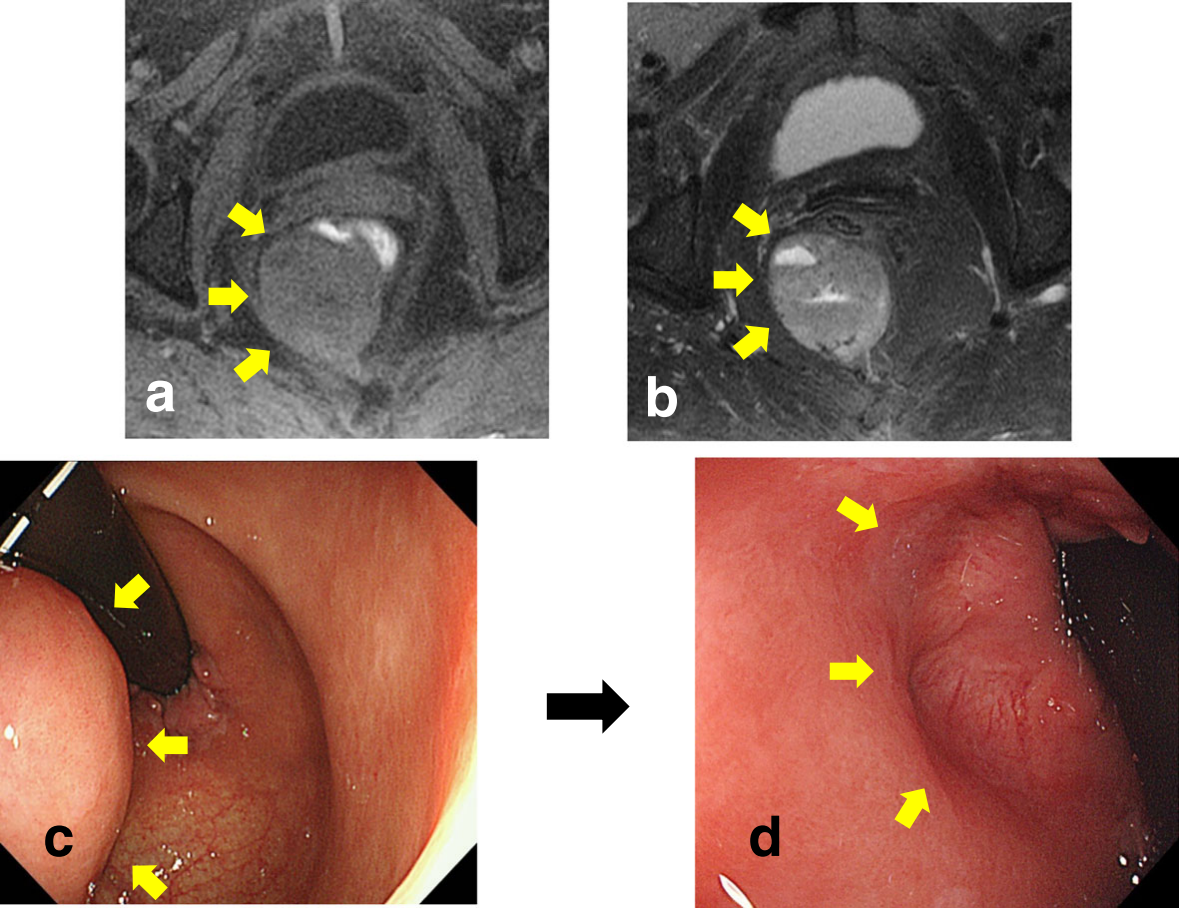
**a** T1-weighted MRI showed a low intensity tumor. **b** T2-weighted MRI showed a high intensity tumor. **c** Colonoscopic examination revealed a submucosal tumor at the posterior rectal wall, and that the distal tumor margin was 1 cm above the dentate line. **d** Colonoscopic examination revealed that the tumor markedly decreased after the treatment of imatinib

A 75-year-old woman with metrorrhagia visited a gynecology clinic. Transvaginal ultrasound showed a retroperitoneal tumor. She was referred to our hospital for a detailed examination. The results of the laboratory examinations were as follows: white blood cell count, 5660/mm^3^ (normal range 4500–9000); red blood cell count, 383 × 10^4^/mm^3^ (normal range 435–555); hemoglobin level, 12.5 g/dL (normal range 13.6–17.0); platelet count, 26.8 × 10^4^/mm^3^ (normal range 14.0–36.0); serum blood urea nitrogen level, 18.6 mg/dL (normal range 8–20); serum creatinine level, 0.46 mg/dL (normal range 0.5–1.2); and tumor marker levels were within the normal ranges (carcinoembryonic antigen, 3.9 ng/mL and CA, 19–9 2.0 U/mL). The laboratory data were within normal limits, instead of demonstrating them. She had no history of serious illnesses, operations, or hospitalizations. Computed tomography (CT) showed an enhanced tumor, measuring 5.3 × 4.2 cm, behind the rectum (Rb) (Fig. 1a and c). Pelvic magnetic resonance imaging (MRI) detected a well-defined tumor with low T1 and high T2 intensities (Fig. 2a, b). Colonoscopic examination revealed that a submucosal tumor at the posterior rectal wall, and that the distal tumor margin was 1 cm above the dentate line (Fig. 2c). Core needle biopsy of the tumor revealed bundles of spindle cells with positive immunohistochemical staining for c-kit and CD34, but negative for S100 proteins. The pathological findings led to the diagnosis of a rectal GIST. Neoadjuvant therapy was initiated with imatinib 400 mg orally daily to reduce the resection range. The therapy was scheduled for 6 months and would be followed by surgical removal of the remaining tumor. CT at 1 month after starting chemotherapy showed that the tumor size had decreased to 4.5 cm. Unfortunately, 4 months after the initiation of neoadjuvant therapy, the patient developed generalized erythematous papules with severe itching. CT examination and colonoscopy revealed that the tumor size had decreased to 3.5 × 3.5 cm (Figs. 1b, d, and 2d). The patient underwent a partial sphincter-saving rectal resection with creation of an ileostomy.

**Fig. 3:**
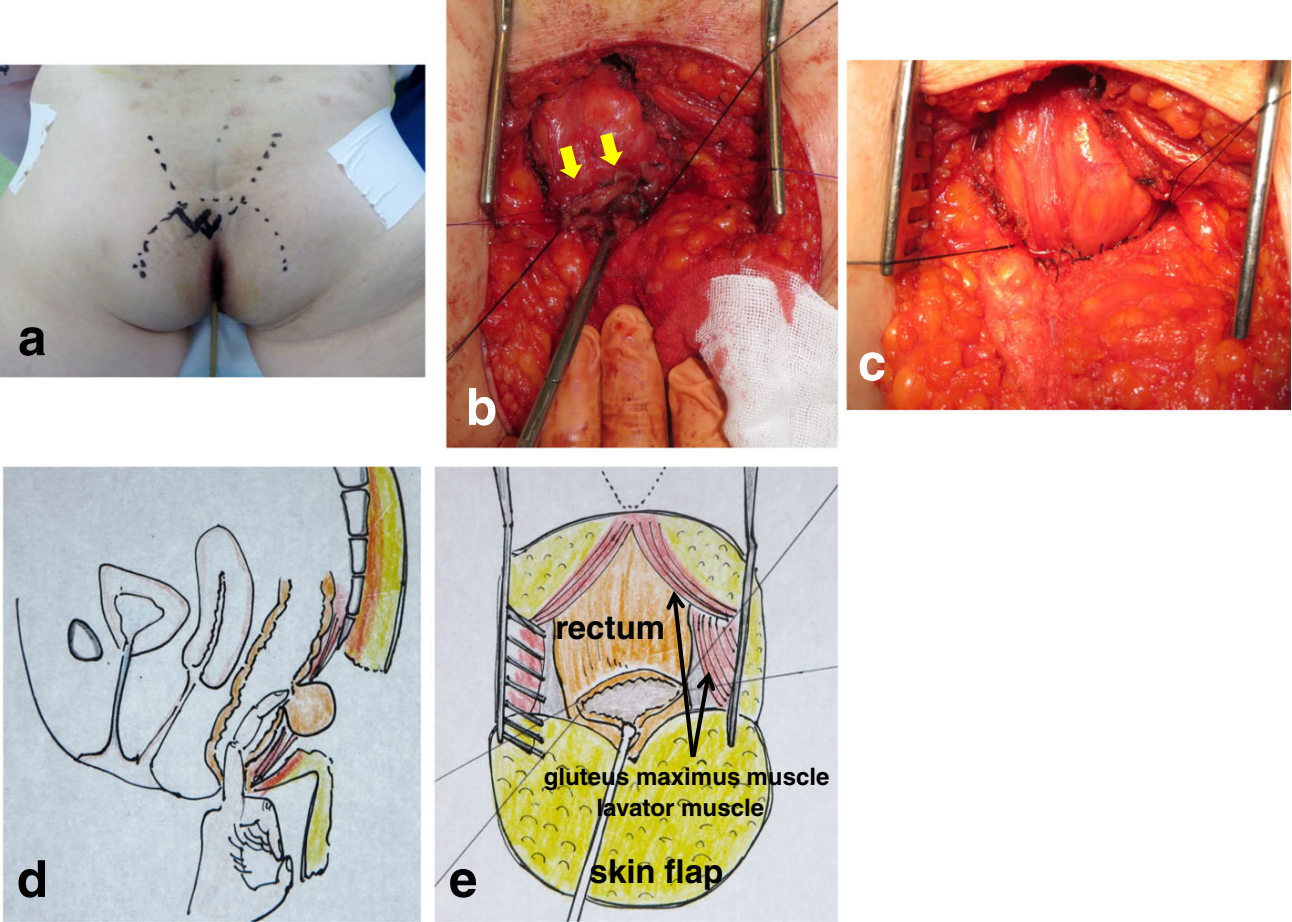
**a** The patient was placed in the jackknife position, with the buttocks parted by adhesive strapping. **b** This is the image of the tumor resection, showing an opening of the rectum (*arrow*). **c** The rectal wall was closed using the Gambee’s method with suturing of the muscle layer. **d** The tumor, lifted via digital rectal examination, was extracted with a safety margin. **e** This is the schema of Fig. 3b

The patient was placed in a prone jackknife position on the operating table, with the legs slightly abducted and the buttocks strapped apart using adhesive tapes from the buttocks to the table (Fig. 3a). First, the skin flap was made by a cluneal arched skin incision between the subcutaneous fat and lavatory muscle. We then approached the posterior rectal wall through the side of the gluteus maximus muscle and lavatory muscle of the anus. The Waldeyer’s fascia was incised to expose the tumor and the bared rectal wall. The tumor, lifted via digital rectal examination, was extracted with a safety margin (Fig. 3b, d, and e). The rectal wall was closed using the Gambee’s method (absorbable suture), with suturing of the muscle layer (3–0 silk) (Fig. 3c). A 19-Fr. soft silastic tube drain was placed near the suture line, and port site closure was completed. A temporary ileostomy was created via laparoscopic surgery.

**Fig. 4:**
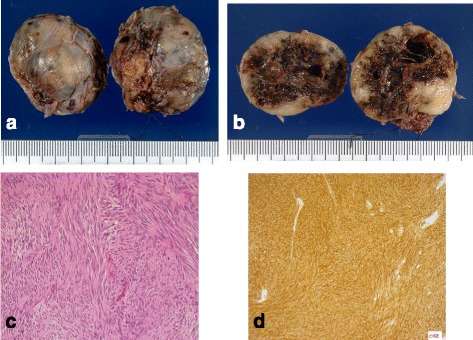
**a**, **b** The resected tumor was 3.5 × 3.5 × 2.5 cm in size. The rubbery-hard tumor with widespread central necrosis was completely capsulized. **c** Microscopic examination (hematoxylin-eosin staining, original magnification: ×400) revealed a formation of spindle-shaped cells. **d** Immunohistochemical staining of the tumor cells for c-kit revealed strong positive findings

**Fig. 5:**
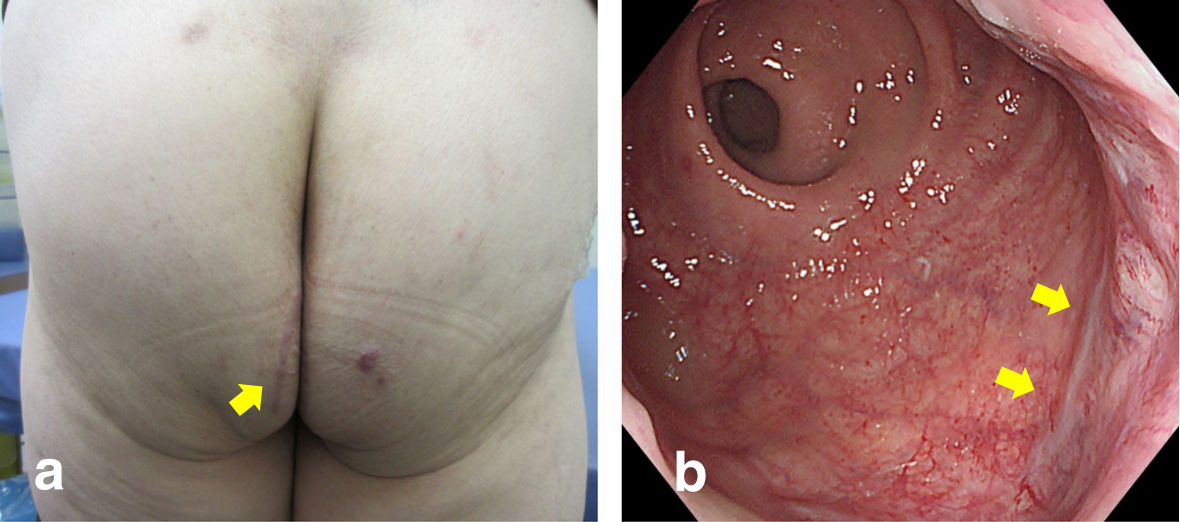
**a** The operative scar was fine and inconspicuous (*arrow*). **b** Colonoscopy was performed before closing the ileostomy. The examination showed a fine scar (*arrow*) with no stenosis

Histopathological examination revealed that the tumor was located in the muscularis of the rectum, with a negative margin (Fig. 4a), and had widespread central necrosis via effective response from imatinib (Fig. 4b). Microscopic examination showed the tumor was consisted of bundle-like proliferations of spindle-shaped cells (Fig. 4c). The immunohistological findings showed that the tumor cells stained negatively with S100 and SMA and positively with c-kit and CD34; the tumor was diagnosed to be a KIT-positive GIST (Fig. 4d). The postoperative course was uneventful, and the ileostomy was closed 2 months later. Postoperative anal function was preserved, and the operative scar was fine and inconspicuous (Fig. 5a and b). Neither local recurrence nor distant metastasis was noted during the 2-year follow-up without adjuvant therapy.

## Discussion

GISTs are rare but are, nevertheless, the most common mesenchymal neoplasms of the gastrointestinal tract. Mazur and Clark first introduced the term GIST in 1983 that constitute around 1% of all primary gastrointestinal cancers [5]. Specific mutations in the so-called KIT oncogene are the most common cause of the development of this tumor [6]. They can be found in the stomach (51%), jejunum and ileum (36%), and colon and rectum (5–7%), but can also occur extra-gastrointestinally in the mesentery or omentum in rare cases [7, 8]. Size and mitotic activity contribute to the risk estimation for “malignant behavior” of GIST, according to the National Cancer Institute (NCI) consensus classification [3, 4, 9]. Surgery remains the therapy of choice for patients with primary GISTs, with no evidence of metastasis and should be the initial therapy if the tumor is technically resectable and associated with an acceptable morbidity risk [3]. The goal of the operation is complete gross resection with a negative microscopic margin (R0 resection) without bleeding and rupture of the pseudocapsule [10]. Neoadjuvant imatinib treatment is also an option to facilitate function-preserving surgeries for tumors in the gastro-esophageal junction and rectum [11, 12]. Imatinib Mesylate is an orally administered competitive inhibitor of the tyrosine kinase associated with the KIT protein (stem cell factor receptor), ABL protein, and platelet derived growth factor receptors. Many studies have shown the effectiveness of imatinib in the treatment of GISTs since its first report in 2001 [13]. As for rectal GISTs, several reports demonstrated that neoadjuvant imatinib treatment improved R0 resection rates and decreased the risk of postoperative morbidity [11, 14]. In the National Comprehensive Cancer Network guidelines, it is recommended that neoadjuvant imatinib treatment should be considered if abdominoperineal resection is necessary to achieve a negative resection margin, or if the surgeon feels that multivisceral resection may be required [15]. However, in the restricted space of the pelvis, complete resection of a large rectal GIST is difficult and often necessitates abdominoperineal resection or intersphincteric resection (ISR), with or without adjacent organ resection. Miettinen et al. reported the treatment of 144 cases of anorectal GIST [2]. In this study, the smaller tumors (≤2 cm) were typically treated by enucleation only, excluding one case. Tumors that were 2–5 cm were also usually treated with local excision. Large tumors (>5 cm) were commonly removed by abdominoperineal or anterior resection with impairment of the sphincter function (15 primary cases and 2 cases for the treatment of recurrence). There are some reports in the literature describing transanal, transcoccygeal, and transvaginal approaches for the local excision of GISTs located in the lower rectum with the aim of decreasing the morbidity rate. Transanal excision is the most minimally invasive approach; however, there is a limit to the distance from the dentate line. Koscinski et al. reported that transanal excision is appropriate for lesions located at an average distance of 3 cm from the dentate line [16]. Furthermore, whether this procedure is possible is often dominated by the physique of the patient. Bleday indicated that transcoccygeal excision was especially useful for lesions at the posterior rectal wall and appropriate for lesions located at an average distance of 5 cm from the dentate line [17]. However, transcoccygeal excision provides a poor field of view because of its high morbidity rate, such as postoperative fistula occurring in 21% of patients [18]. The choice of local resection that preserves the anal function must result in a negative margin different from the conventional extend operation.

In our case, the 5.3-cm sized tumor situated at the posterior wall of the lower rectum and the distal tumor margin was 1 cm above the dentate line. Neoadjuvant imatinib therapy was scheduled for 6 months as the references which reported, in randomized clinical studies, the cumulative incidence of response almost reached a plateau after treatment for 6–8 months, and disease progression occurred in some patients even in this period [19, 20]. After the administration of imatinib, the tumor size decreased to 3.5 cm. The possible operations for selection were abdominoperineal resection, ISR, or local resection. However, we avoided abdominoperineal resection and ISR to preserve her anal function. The tumor was too large to select the transanal approach for local resection. The typical operation using the posterior approach for rectal tumors is the method reported by Kraske [21]. This method has a straight-line incision because the surgical field view is poorer than that of ours. On the other hand, the surgical field view is clear with anal dysfunction by cutting the sphincter in Mason’s method [22]. Finally, we chose the sphincter-saving operation using a cluneal arched skin incision, which has already been reported in Japanese literature [23]. In this case, we performed the operation on the basis of this literature [23]. It is easy to approach the puborectal muscle and external anal sphincter by making a skin flap with an arch-shaped incision at the buttocks. After splitting both sides of the posterior rectum, the tumors lifted from the inside of the rectum were resected with the safety margin. The advantages of this method include preserving the anal function and providing a clear view during the operation. In addition, surgical site infection is prevented as the skin incision in our method is far from the anus.

## Conclusions

We describe our experience with the patient who underwent a novel approach that improves the operative field visibility in resecting a tumor with a safety margin and preserves a patient’s anorectal sphincter function.

## Abbreviations

CT: Computed tomography
GISTs: Gastrointestinal stromal tumors
ISR: Intersphincteric resection
MRI: Magnetic resonance imaging
NCI: National Cancer Institute
